# Perceptions and knowledge of air pollution and its health effects among caregivers of childhood cancer survivors: a qualitative study

**DOI:** 10.1186/s12885-021-08739-y

**Published:** 2021-09-30

**Authors:** Austin R. Waters, Echo L. Warner, Perla L. Vaca Lopez, Anne C. Kirchhoff, Judy Y. Ou

**Affiliations:** 1 Cancer Control and Population Sciences, Huntsman Cancer Institute, 2000 Circle of Hope, Salt Lake City, UT 84112 USA; 2 University of Arizona Cancer Center, Tucson, AZ USA; 3 College of Nursing, University of Arizona, Tucson, AZ USA; 4 Department of Pediatrics, University of Utah, Salt Lake City, UT USA

**Keywords:** Air pollution, Childhood Cancer survivor, Late effects, Cancer caregiver, Information seeking, Exposure reduction

## Abstract

**Background:**

Emerging research suggests that survivors of childhood and adolescent cancers are at risk for morbidity and mortality associated with air pollutants. However, caregiver perceptions of the effects of air pollution are unknown. Thus, to address this gap we described caregivers’ perceptions of air pollution’s impact on general population health and specifically on childhood cancer survivors, and caregivers’ air pollution information-seeking and exposure reduction behaviors.

**Methods:**

Participants were Utah residents, ≥18 years, and caregiver of a childhood cancer survivor who had completed treatment. Semi-structured interviews were conducted with caregivers to describe their perspectives on air quality, how air pollution impacts health (general population and survivor health), and their information seeking and exposure reduction behaviors. Interviews were recorded, transcribed, and analyzed through two rounds of structured coding.

**Results:**

Caregivers (*N* = 13) were non-Hispanic white and primarily females (92.3%) between 30 and 49 years old (46.2%). Most families lived within the Wasatch Front (69.2%), the main metropolitan of Utah. Two categories emerged pertaining to caregiver’s perceptions of air pollution: 1) Limited awareness about the health effects of air pollution, and 2) Unsuccessful information seeking and minimal exposure reduction behaviors. All caregivers held negative perceptions of air pollution in Utah, but most were unaware of how pollution affects health. While some families limited air pollution exposure by avoiding outdoor activity or physically leaving the region, few practiced survivor-specific exposure reduction. Nearly half of caregivers worried about potential effects of air pollution on survivor health and wanted more information.

**Conclusions:**

Despite negative perceptions of air pollution, caregivers were divided on whether air pollution could impact survivor health. Few caregivers engaged in exposure reduction for their cancer survivor. As air pollution levels increase in the U.S., continued research on this topic is essential to managing cancer survivor respiratory and cardiovascular health.

**Supplementary Information:**

The online version contains supplementary material available at 10.1186/s12885-021-08739-y.

## Background

Cancer survivors represent approximately 5% of the United States population [1], with an estimated 500,000 survivors of childhood cancer in the United States (U.S.) [2]. The numbers of childhood cancer survivors are growing due to vast improvement in cancer treatments over the past few decades, but these improvements come at a cost. Because of the toxicity of their cancer treatment, survivors of childhood cancers are at high risk for developing lung health conditions including asthma, respiratory infections, and pulmonary fibrosis (late effects) [3, 4]. Further, chronic respiratory diseases among childhood cancer survivors that reach young adulthood are so common that they are comparable to the prevalence in frail, elderly populations [5, 6].

Air pollution is a major public health concern in the U.S. and may disproportionately affect mortality and morbidity among cancer survivors [7–9]. Fine particulate matter air pollution (PM_2.5_) is a pollutant of major of concern for children in general and adults with lung conditions [10, 11]. Emerging research supports that PM_2.5_ is associated with an increased risk for mortality and respiratory hospitalizations among childhood cancer survivors [7, 12, 13], whose risk for respiratory hospitalization is significantly higher compared to cancer-free persons. The risk is also the highest for survivors who were treated with chemotherapy, suggesting that therapy-related toxicity may sensitize lung tissue to air pollutants [12].

The environment in which a cancer survivor lives plays a significant role in their survival and severity of their post-treatment morbidity [12]. Caregivers of childhood cancer survivors do seek information about how environmental risk factors may affect their child’s cancer diagnosis. In a national survey, 88% of pediatric oncology providers reported questions from caregivers about the associations between the environment and their child’s cancer, although these inquiries primarily focused on the etiology of cancer [12]. Because of the newness of the emerging literature and a lack of clinical guidance on this topic, caregivers of childhood cancer survivors may not consider air pollution to be a health risk for their child post cancer therapy. Understanding how caregivers perceive the role of air pollution on survivor health can help inform the direction of new studies and identify whether educational interventions are necessary.

At the policy level, despite growing evidence of the health effects of air pollution on individuals with cancer [8, 9], public health agencies do not define cancer survivors as a population that should consider avoiding pollution exposure. Additionally, deregulation and decreases in Clean Air Act enforcement since 2016 have resulted in an increase in premature mortality in the general population due to higher levels of particulate matter [14], which potentially has devastating health impacts on susceptible populations, such as childhood cancer survivors. Thus, a better understanding of cancer caregivers’ perceptions and knowledge of air pollution could help to shape clinical guidance for families experiencing cancer and to inform future public health policy as evidence on this topic continues to emerge [7, 12],

We conducted semi-structured qualitative interviews with caregivers of childhood cancer survivors in Utah’s main metropolitan areas, which consistently rank among the most polluted cities in the U.S., for short-term PM_2.5_ [13, 15]. In fact, in 2017, 2018, and 2019, counties in Utah with air monitors had a cumulative total of 128 days exceeding 35 μg/m^3^, far surpassing the national air quality standard set to allow 2 % of the days, or roughly 21 days, during the 3 years to exceed 35 μg/m^3^ [13]. PM_2.5_ tends to be higher in the winter due to temperature inversions and has been documented as high as 132.5 μg/m^3^ in Salt Lake County during cold weather conditions [16]. In addition to wintertime PM_2.5_ Utah is experiencing increases in summer PM_2.5_ due to wildfires [17]. Up to 80% of Utah’s population lives in counties with the worst short-term PM_2.5_ pollution in the state [18, 19]. Our goal was to describe caregivers’ perceptions of air pollution and its impact on health (both general and survivor-specific) as well as any experiences with information seeking and exposure reduction behaviors.

## Methods

### Study setting

Due to Utah’s topography and weather patterns, extreme short-term exposure to air pollution from winter inversions and summer wildfires is common. As of 2019, an estimated 75% of Utah’s 3.22 million residents live in four (Salt Lake, Utah, Davis, and Weber) of the state’s 29 counties [20]. The metropolitan areas within these four counties are surrounded by the Wasatch Mountains, and are commonly referred to as the Wasatch Front. The Wasatch Mountain range circles around valleys, creating bowl-like topography. During the winter months, the cold air traps pollution in the valley. During the summer, wildfire smoke from fires in Utah and surrounding states flows into the Wasatch Front. Located in the Wasatch Front is Primary Children’s Hospital (PCH), the only pediatric oncology center in Utah where most pediatric cancer patients in the region are treated [21]. It is common for families receiving treatment at PCH to reside outside of the Wasatch Front, although most spend substantial amounts of time at PCH during their child’s cancer treatment.

### Participants and data collection

Eligible participants were 18 years or older, residents of Utah, and the caregiver of a childhood cancer survivor who completed treatment at PCH. Potential participants who met these eligibility criteria were either 1) identified through a pre-established cohort of participants who agreed to be contacted for future research, or 2) caregivers whose child had post-treatment follow-up appointments at PCH. Caregivers participated in the informed consent process, completed a brief survey of caregiver and survivor demographics and treatment information, and took part in a semi-structured telephone interview conducted by two members of the research team (JO or ARW).

An iterative and cyclical approach was taken during interview data collection and analysis. As such the concept of data saturation was used to guide decisions on the number of interviews conducted [22]. The iterative and cyclical approach also included interview script modification to facilitate a dive deeper into emergent categories as the topic of inquiry has not been qualitatively described prior. The script was developed for this study and asked questions about caregivers thoughts on 1) The environment including: air quality in Utah, how air pollution impacts general population health, information seeking, and risk reduction behaviors, as well as more specific probes about 2) The environment in relation to cancer including: how air pollution impacts survivor health, information seeking about the environment in relation to survivor health, and any survivor specific risk reduction behaviors (Supplement 1). Interviews lasted between 10 and 22 min, with the median length being 15 min. Each participant received a $10 gift card as a thank you for their time. All study activities took place in English between October 2018 and May 2019 and were approved by the University of Utah Institutional Review Board (IRB#00111463).

### Data analysis

To characterize the study population descriptive statistics were calculated for survivor and caregiver demographics using STATA 14 (StataCorp, College Station, Texas). Interviews were recorded, transcribed, and quality checked with the audio files to rectify discrepancies. Qualitative data were analyzed using qualitative description [23] via two cycles of structured coding to generate categories and subcategories using NVivo 11 (QSR International, Doncaster, Australia) [24]. During first-cycle coding, three members of the research team (ARW, PVL, JO) read and employed initial coding on a total of 20% of the interviews to capture first impressions. Roundtable discussion was then used to begin categorization of codes to build the initial NVivo coding scheme. Then, an additional 20% of the interviews were coded sentence by sentence to further refine the coding scheme. During second-cycle coding, an additional 20% of the interviews were double coded by ARW and JO using focused coding, a technique that searches for the most frequent or salient ideas and categorizes them based on thematic similarity [24]. Focused coding was used during second-cycle coding to compare and categorize the codes. Focused coding was compared between ARW and JO; codes with zero agreement were discussed and resolved by coder consensus, resulting in a high level of agreement (κ = 92.0) [25]. Coding decisions and interpretations were discussed by ARW, JO, and ELW throughout the process. This coding resulted in two main categories including: 1) Limited Awareness about the Health Effects of Air Pollution and 2) Unsuccessful Information Seeking and Minimal Exposure Reduction Behaviors. Each category included sub-categories developed from both questions about the environment exclusively and more specific probes about the link between the environment and cancer survivor health.

## Results

### Caregiver and childhood cancer survivor demographics

Of the 19 caregivers who were contacted, 13 participated in the study activities (68.4%). The other six were unable to be contacted for the interview after initial contact. Participating caregivers were all non-Hispanic White and primarily females between 30 and 49 years of age (Table 1). Over half of participants had a college/technical degree or higher (69.2%) and nearly 40% reported an annual household income of $75,000 or more. Caregivers reported that 84.6% of children were diagnosed with cancer between 0 and 4 years of age, with over half aged between 5 and 10 years at time of study (53.8%). All of the survivors underwent chemotherapy during their cancer treatment (100%), while a minority had radiation (23.1%) and/or surgery (23.1%). Most families lived within the Wasatch Front (69.2%).

**Table 1:** Caregiver and Survivor Sociodemographic Characteristics

	N	%
Caregiver characteristics (*N* = 13)		
Gender		
Female	12	92.3
Male	1	7.7
Race/Ethnicity		
Non-Hispanic White	13	100.0
Age at interview, years		
20–29	2	15.4
30–39	6	46.2
40–68	5	38.5
Education		
Some high school	1	7.7
Some college/technical school	3	23.1
College/technical school graduate	7	53.8
Graduate degree	2	15.4
Annual household income		
Less than $25,000	1	7.7
$25,000 - $49,999	1	7.7
$50,000 - $74,999	3	23.1
$75,000 - $99,999	5	38.5
$100,000 or more	3	23.1
Cancer survivor characteristics (*N* = 13)		
Age at cancer diagnosis, years		
0–4	11	84.6
5–10	2	15.4
Age at caregiver interview, years		
0–4	3	23.1
5–10	7	53.8
11–15	2	15.4
16–18	1	7.7
Type of cancer treatment^a^		
Chemotherapy	13	100.0
Radiation	3	23.1
Surgery	3	23.1
Family lives in the wasatch front		
Yes	9	69.2
No	4	30.8

^a^ Some survivors received more than one kind of treatment; therefore, total percentages exceed 100%

### Category: limited awareness about the health effects of air pollution

Under this category, three sub-categories emerged. The first focused on perceptions of air quality: 1) Air quality is *“terrible.”* Almost all caregivers expressed very negative perceptions of the air quality in the Wasatch Front. The second sub-category emerged when we asked caregivers about their perceptions of air quality on health in general: 2) Limited understanding of how air pollution effects health. In this category, uniformly negative perceptions of air pollution in Utah were not indicative of knowledge about the health impacts of air pollution on the general population. When asked specifically if they were aware of a link between air pollution and childhood cancer survivors’ health the third sub-category emerged: 3) Mixed feelings regarding how air pollution affects childhood cancer survivors.

#### Sub-category: air quality is “*terrible*”

When caregivers were asked about their perceptions of the environment in Utah – specifically air pollution – almost every caregiver expressed very negative perceptions of the air quality, especially those who lived in the Wasatch Front. The air quality in Utah was commonly described as *“very bad,”* with one caregiver who said, *“It’s horrible, the air pollution is absolutely horrible.”* It was fairly common for caregivers to express their dissatisfaction with the air quality and feelings of being unable to escape it. One caregiver who lived in the Wasatch Front stated, *“We just feel trapped.”*

#### Sub-category: limited understanding of how air pollution affects health

When asked about the possible impacts of air pollution on health in general, only a few caregivers believed air pollution likely had negative effects on general population health. One caregiver who did perceive a connection shared, *“You can’t breathe in garbage [air pollution] all day and not [have health problems] eventually.”* Caregivers more commonly described that air pollution primarily affected vulnerable populations such as the elderly, children, and those with pre-existing respiratory conditions (e.g. asthma). One caregiver was somewhat uncertain, however, stating, *“I don’t know … but I could see air pollution affecting kids for sure.”*

### Sub-category: mixed feelings regarding how air pollution affects childhood cancer survivors

When probed about the connection between air pollution and survivors’ health, nearly half of all caregivers reported concern. One caregiver stated: *“Well, I don’t know if it is [connected], but I worry [it is].”* Few caregivers were able to report why or how air pollution might negatively impact their child’s health, although one caregiver stated, *“I think it can for sure, especially if they already had respiratory issues because of their treatment, it can be exacerbated by the air quality for sure.” *This concern about survivors’ lung health originated from the caregiver’s awareness of respiratory late effects. Another caregiver shared a similar view stating, *“The biggest thing I’m worried about is the respiratory stuff.”*

The remaining half of caregivers reported that they felt air pollution had no detrimental effects on survivor health. When asked if they thought air pollution could impact survivor health, one caregiver’s response summed up the sentiment: *“No, not necessarily.”* Regardless of whether caregivers felt that air pollution could affect vulnerable populations, it was not a uniformly held perception that cancer survivors were a population susceptible to negative health effects associated with air pollution. Caregivers who were not concerned about air pollution implied that they would already be informed of such an association if it existed: *“I don’t [think there is a connection], I have never heard of anything of the kind.”*

### Category: unsuccessful information seeking and minimal exposure reduction behaviors

While caregivers reported having minimal knowledge about the connection between air pollution and survivor health, this knowledge deficit motivated some of them to seek information about air pollution’s effect on health. However, for most caregivers, this did not lead them to engage in any behaviors that would reduce the survivors’ exposure to air pollution.

Our first sub-category describes: 1) Information seeking about the environment and survivor health. Some caregivers reported seeking but not finding information on air pollution and survivor health. When caregivers were unable to find information about air pollution’s effect on health in general, they assumed there was no link. This led to limited enactment of exposure reduction behaviors. The exposure reduction that caregivers engaged in was almost exclusively on the familial level and was driven by caregivers’ negative perceptions of air pollution rather than a fear of health effects. Thus, resulting in our second sub-category: 2) Minimal use of exposure reduction behaviors. Exposure reduction behaviors were not commonly practiced by most caregivers and subsequently their families. Finally, when we asked specifically about exposure reduction behaviors for their childhood cancer survivor, only three caregivers took action to reduce exposure specifically for their child with a cancer history. Our final sub-category: 3) Infrequent survivor-specific exposure reduction.

#### Sub-category: information seeking about the environment and survivor health

When probed about information seeking pertaining to the topic of the environment and survivor health, some caregivers reported that they had become more aware and curious about the environment’s impact on health since their child’s cancer treatment, primarily being immunocompromised during chemotherapy. The small minority of caregivers who found information on this topic found it on social media, from local news articles, and academic research. While some caregivers reported seeking information about air pollution and survivor health, no caregivers reported asking their child’s oncologist or any other health care professional about the health effects of air pollution. When discussing this topic, caregivers expressed a need for more information, specifically air pollution’s impact on survivor health. For example, one caregiver shared their preferences, saying, *“Information, like up and coming [research], something about environmental [exposure] … things that would negatively affect the health of my daughter or any kids that are post-cancer.”*

#### Sub-category: minimal use of exposure reduction behaviors

Despite a general lack of knowledge about the potential negative effects of air pollution on general population health, some caregivers reported frequently taking measures to reduce their family’s exposure to air pollution. These behavioral changes were driven by negative perceptions about air pollution itself and not their concern about air pollution’s health effects. This included reducing time spent outdoors when they perceived air pollution as worse than usual. Most caregivers who were concerned about air pollution reported checking air quality that day by visual assessment (i.e. looking outdoors or walking outdoors).

One common exposure reduction behavior was leaving the Wasatch Front when they perceived the air quality was poor, usually during times of a winter inversion: *“We went outta town a lot, and we just didn’t really do much activity outside.”* One caregiver also shared that they considered moving out of the state to avoid the poor air quality, stating, *“I wish I could afford to move, but right now we can’t afford to move.”* Additionally, caregivers who did not live in the Wasatch Front often mentioned air quality as one of the reasons why they live outside of the area. One caregiver shared, *“We refuse to live there [Wasatch Front] because of the air quality, specifically.”* Caregivers who felt a strong urge to avoid air pollution by moving or vacationing out of the state were not motivated to do so because of their child with cancer’s health, but rather due to negative perceptions of air pollution itself.

#### Sub-category: infrequent survivor-specific exposure reduction

We probed further to understand if exposure reduction behaviors among caregivers’ families were similar to the families’ other children or if they were different for their child who had cancer. We found that most caregivers did not attribute their exposure reduction behaviors to their child being a cancer survivor. Their exposure reduction behaviors for the child with a cancer history also did not differ from the child’s other siblings. This was primarily because caregivers held the belief that they should not impede on the survivor’s childhood. One caregiver summed up this sentiment by stating, *“We saved her life so she could live.”* Caregivers living outside the Wasatch Front did not report any survivor specific exposure reduction behaviors. One caregiver who lived in a city outside the Wasatch Front stated, *“I feel very confident with her playing outside here.”*

There were 3 caregivers living in the Wasatch Front who reduced their child’s exposure to air pollution specifically because of their child’s history with cancer and their past experiences with immunosuppression. These caregivers reported keeping their child indoors during poor air quality days and installing air filters in the home. *“He has permission from his school to stay inside during bad air days … and I have an air filter in my house that we run,”* stated one caregiver.

## Discussion

We found that caregivers of childhood cancer survivors uniformly held negative perceptions of air pollution. However, most caregivers were unsure how air pollution affects public health and reported a desire for information about how the environment could affect their child’s health after cancer treatment ends. Some caregivers reported seeking survivor-specific environmental health information but were often unsuccessful in their search, as the majority of the current research focuses on cancer etiology. Families reduced exposure by avoiding being outdoors during days with poor air quality. These behaviors, however, were often informed by visual assessment of pollution and were not motivated by avoidance of potential health impacts, rather their negative perceptions of air pollution. Additionally, exposure reduction specifically for cancer survivors was practiced by very few caregivers, even though nearly half of caregivers reported being concerned about how air pollution could affect survivor health.

Caregivers were often unaware of the risks that air pollution posed to health, which suggests limited public awareness of the adverse health effects of air pollution. While not surprising, these findings are crucial in beginning to understand caregivers’ perceptions and future responses to air pollution as emissions of air pollutants increase across the U.S. due to climate change, the threat of deregulation of pollution emissions increases in climate-related summer wildfire smoke, and the possibility of reductions in state and federal agencies’ capacity to enforce the Clean Air Act [14, 26]. Risk perception literature suggests that individuals must first understand a risk exists and then begin to understand how that risk applies to them through lived experiences before they can take action to accordingly change their behavior [27, 28]. Some caregivers who brought up concerns about air pollution’s impact on survivor health were already aware of respiratory late effects and connected the idea that their child breathing in air pollution could impact their child’s respiratory health. Caregivers who best described the potential risk of air pollution on survivor health made this connection due to the experience of their child being immunocompromised during cancer treatment. These caregivers felt their experience with their child’s compromised immunity during cancer treatment was similar to the way other environmental risk factors, like air pollution, could affect their child’s health post-treatment. In both situations, they were motivated to engage in risk reduction behaviors to protect their child.

Caregivers’ awareness of the health effects of air pollution prior to their child’s cancer diagnosis appears to be key to their understanding of the potential risk that air pollution posed to their child after cancer treatment ended. Our findings suggest that in highly polluted areas, like the Wasatch Front, caregivers of childhood cancer survivors could benefit from air pollution focused public health campaigns as many did not understand the link between air pollution and general public health. Campaigns with multiple methods of dissemination (e.g., social media marketing, classroom and community based education, etc.) may have the potential to increase awareness of health effects of air pollution and begin to foster risk formation in the general population [29]. For example, caregivers in our study often held the assumption that if such associations existed this information would be widely disseminated through media or other sources. Unlike the existing literature, which demonstrates that media sources, health care providers, and information distributed by local health departments are the top sources for environmental health information [30], none of the caregivers we interviewed had discussed environmental impacts on survivorship or late effects with their providers or obtained information from health departments. Other strategies for expanding educational opportunities about environmental impacts on health among cancer patients and survivors should also be explored.

Caregivers in our study and in the existing literature [31] expressed the need for more information about the effect of environmental exposures on childhood cancer survivors, both in how environmental pollutants may have influence the cancer’s original growth and how they affect health after treatment ends. However, caregivers and patients often see oncology providers as a trusted source to provide education on survivor health and late effects [32, 33], meaning that oncology providers may play a key role in dissemination of high-quality information about how childhood cancer survivor health may be impacted by the environment.

Understanding the associations between air pollution and survivor health outcomes are a new area of research, but a growing body of studies demonstrate that air pollution does lead to negative health outcomes for this population [7–9, 12]. Thus, at this nascent stage, clinical survivorship guidelines do not currently include air pollution education [34], nor are oncology providers currently trained in the fields of environmental health or environmental risk assessment. Further research on environmental pollutant exposure among cancer survivors is needed to establish an evidence base that outlines clinical guidance to equip providers and educators with tools to discuss environmental risk factors like air pollution with cancer survivors and their caregivers [31]. This study suggests a potential opportunity for further survivor-focused environmental research to be conducted and incorporated into clinical management of childhood cancer survivors. The incorporation of environmental exposures in clinical care is uncommon. However, emerging literature in the field of symptom science in nursing [35], advocates for the integration of the impacts of environmental exposure on symptom management into the National Institutes of Health Symptom Science Model [35].

Our results also demonstrate that caregivers engage in a variety of exposure reduction behaviors including limiting their family’s daily activities and staying indoors, opting out of living in the Wasatch Front, and being motivated to relocate their families to less polluted areas. Such migration has been observed in populations of other highly polluted areas such as China and Germany [36, 37]. While local air quality tracking systems are available in the Wasatch Front, caregivers in our study often reported visually checking air quality by looking outside when deciding if they should reduce exposure to air pollution. Alerts from air quality tracking systems increase awareness of air quality [38]; however, increased awareness of air quality has mixed efficacy on actual exposure reduction [38–40]. Our results suggest that even if caregivers had a higher awareness of air pollution’s effects on survivor health, only so much can be done by an individual to escape their environment and that behavior change in conjunction with policy changes may be more efficacious in protecting the health of childhood cancer survivors [41].

### Limitations

Our study has a few notable limitations pertaining to our sample and study design. While our sample does reflect Utah demographics, which is largely white, our sample lacked representation of racial and ethnic minorities. As air pollution is more commonly perceived by individuals of color as a health hazard and these individuals are more likely to live in communities with high levels of pollutants, data from racial and ethnic minority caregivers is a priority for future studies [42, 43]. Our study included more caregivers from the Wasatch Front than less populous counties in Utah. Therefore, the perceptions and behaviors of rural participants may not be equally represented, although the majority of the population in Utah does live along the Wasatch Front. Our study described the perceptions of caregivers but did not interview oncologists, who provide survivorship care and education on risk factors and have insights that may strengthen the integration of environmental health into survivorship care. Further, our study was cross-sectional and conducted partly during the winter months, which usually have worse air quality than other seasons, and could have heightened awareness of environmental pollutants and skewed perceptions to be more negative. Lastly, due to the novel nature of the topic, being a first step towards understanding perceptions of air pollutions impact on survivors, qualitative description and a categorical display of results were used [23, 44]. Interview times in this study were short due to participant’s general lack of awareness and unsuccessful information seeking about the impact of PM_2.5_ on cancer survivor health. However, what is not being said or awareness that is not present is equally as important as in-depth understanding which may be present in studies with longer interview times. Future inquiry on similar topics should consider priming potential participants with formative findings, such as this study, prior to interviews to achieve further depth and move towards theoretical development. Despite these limitations, the findings presented here are a novel description of caregivers’ perceptions of air pollution and its impact on cancer survivor health, an understudied topic.

## Conclusion

Almost all caregivers of childhood cancer survivors in this study shared negative perceptions of the air quality on the Wasatch Front, Utah. Most lacked an understanding of how air pollution could impact general population and survivor health. Nearly half of caregivers expressed concern about how air pollution could impact their childhood cancer survivor. Some caregivers practiced information seeking about the impact of air pollution on survivor health, but uncommonly reduced their child’s exposure to air pollution. Caregivers desire education on the impact of environmental exposure on late effects. Individual exposure reduction behavior change alone may not be the most efficacious way to reduce survivor exposure to air pollution. Rather public health campaigns and policy changes in conjunction with individual exposure reduction behaviors may serve as a more effective starting point to mitigate the emerging associations of air pollution on survivor health. Additionally, due to the emergent nature of this area of research, the health effects of air pollution have not yet been incorporated into survivorship guidelines or as a part of the definition of susceptible populations, despite the fact that 80% of the U.S. population lives in areas that have air pollution problems. Our study indicates that risk of environmental exposures after cancer treatment ends should be studied in depth and may contribute to important recommendations for cancer survivorship care.

## Supplementary Information

**Additional file 1.**

## Data Availability

Not applicable.

## Abbreviations

U.S.: United States
PM2.5: Fine Particulate Matter
PCH: Primary Children’s Hospital
