# Supplementary material for: Perceptions and knowledge of air pollution and its health effects among caregivers of childhood cancer survivors: a qualitative study
Source: BMC Cancer. 2021 Sep 30;21:1070. doi: 10.1186/s12885-021-08739-y (PMC8482574; doi:10.1186/s12885-021-08739-y)
Supplement: Supplementary file 1 — Additional file 1. [file 12885_2021_8739_MOESM1_ESM.docx]

**Supplement 1: Interview Guide**

**The Environment**

This next section of questions is going to be more generally about the environment such as the outdoors, houses, and schools. We will ask about your perceptions of the link between the environment and the health of the general population as well as cancer survivors.

1. How would you rate the air here in Utah?

2. Do you think that air pollution affects the health of general public?

If yes, why?

If no, why not?

3. Utah has days called “Bad Air Days.” On a Bad Air Days do you change where or when your child goes outside?

If yes – ask for more details

If no – ask why not

4. Do you have other children? (ask if they haven’t mentioned yet)

If yes – ask: On a Bad Air Day, do you treat your child who survived cancer differently than your other children?

If no – Okay we will just move along

5. Does your child’s school have a “Red Air” or bad air day policy?

6. Any other thoughts or comments that you think it would be important for us to know in regards to recurrence, late effects or the environment?

**Recurrences**

Script: Cancer can come back. This is called a recurrence or relapse. The next questions ask about your perceptions of cancer recurrence.

1. Are you concerned about a cancer recurrence in your child who had cancer? Yes No

2. Do you think there are any actions that parents can take to prevent a recurrence?

If yes, what are they? What do you do?

If no, why not?

3. Do you think that recurrence can be influenced by factors in the environment such as the outdoors, homes, and schools ? (If needed, can list examples of factors like air, water, soil, germs, etc).

If yes, explain how so?

If no, why?

**Late effects**

Script: Children who survived cancer may develop long-term health problems called late effects. Like side effects, these late effects are related to the cancer therapy. Unlike side effects, late effects start after treatment ends. The next questions are about late effects.

1. Prior to this interview, had you ever heard of late effects? Yes no

If Yes, could you name a couple of them?

2. Are you concerned about late effects for your child? Yes No

3. Did your oncologist or any other health care provider talk to you about late effects? Yes No

All open ended questions:

| If yes… | If no… |
| --- | --- |
| If yes, who had the conversation with you? | If no – which healthcare provider would you have liked to have had a discussion about late effects with? |
| If yes, when did they talk to you about late effects? | If no - what time would have been the best time to have the discussion? |
| Was that the best time to have a discussion about late effects? |  |
| If yes, what did they tell you about late effects? | If no – what information do you wish a healthcare provider told you about late effects? |
| If Yes, how important do you think it is for parents to be told about late effects?  Very important  Somewhat Important  Neutral  Not important | If no – How important do you think it is for parents to   be told about late effects?  Very important  Somewhat Important  Neutral  Not important |

4. This is another question about the environment. Do you think that late effects can be influenced by factors in the environment such as the outdoors, homes, or schools?

Yes No

If yes, why?

- if mention air pollution, probe and skip question 3

If no, why?

5. Where have you sought information about air pollution? Air pollution and its relation to cancer?

6. Do you think air pollution has any impact on the severity of late effects among childhood cancer survivors?

If yes, why?

If no, why not?
